# Hailstones: A Window into the Microbial and Chemical Inventory of a Storm Cloud

**DOI:** 10.1371/journal.pone.0053550

**Published:** 2013-01-23

**Authors:** Tina Šantl-Temkiv, Kai Finster, Thorsten Dittmar, Bjarne Munk Hansen, Runar Thyrhaug, Niels Woetmann Nielsen, Ulrich Gosewinkel Karlson

**Affiliations:** 1 Department of Environmental Science, Aarhus University, Roskilde, Denmark; 2 Microbiology Section, Department of Bioscience, Aarhus University, Aarhus, Denmark; 3 Stellar Astrophysics Centre, Department of Physics and Astronomy, Aarhus University, Aarhus, Denmark; 4 Max Planck Research Group for Marine Geochemistry, Institute for Chemistry and Biology of the Marine Environment, University of Oldenburg, Oldenburg, Germany; 5 Department of Biology, University of Bergen, Bergen, Norway; 6 Danish Meteorological Institute, Copenhagen, Denmark; Uppsala University, Sweden

## Abstract

Storm clouds frequently form in the summer period in temperate climate zones. Studies on these inaccessible and short-lived atmospheric habitats have been scarce. We report here on the first comprehensive biogeochemical investigation of a storm cloud using hailstones as a natural stochastic sampling tool. A detailed molecular analysis of the dissolved organic matter in individual hailstones via ultra-high resolution mass spectrometry revealed the molecular formulae of almost 3000 different compounds. Only a small fraction of these compounds were rapidly biodegradable carbohydrates and lipids, suitable for microbial consumption during the lifetime of cloud droplets. However, as the cloud environment was characterized by a low bacterial density (Me = 1973 cells/ml) as well as high concentrations of both dissolved organic carbon (Me = 179 µM) and total dissolved nitrogen (Me = 30 µM), already trace amounts of easily degradable organic compounds suffice to support bacterial growth. The molecular fingerprints revealed a mainly soil origin of dissolved organic matter and a minor contribution of plant-surface compounds. In contrast, both the total and the cultivable bacterial community were skewed by bacterial groups (γ-*Proteobacteria*, *Sphingobacteriales* and *Methylobacterium*) that indicated the dominance of plant-surface bacteria. The enrichment of plant-associated bacterial groups points at a selection process of microbial genera in the course of cloud formation, which could affect the long-distance transport and spatial distribution of bacteria on Earth. Based on our results we hypothesize that plant-associated bacteria were more likely than soil bacteria (i) to survive the airborne state due to adaptations to life in the phyllosphere, which in many respects matches the demands encountered in the atmosphere and (ii) to grow on the suitable fraction of dissolved organic matter in clouds due to their ecological strategy. We conclude that storm clouds are among the most extreme habitats on Earth, where microbial life exists.

## Introduction

Airborne bacteria have lately generated a lot of interest, due to their ubiquitous presence and the accumulating evidence of their activity in the atmosphere [1]. Previous studies indicate that terrestrial habitats, in particular soils and plant leaf surfaces, are the major sources of airborne bacteria, whereas marine environments are a less prominent source [2]. By performing a meta-analysis of the composition of the airborne community and of their potential source environments, Bowers et al [3] identified bacterial taxa indicative for soil and plant-surface origin. Generally, they found that the airborne community was more similar to plant-surface than to soil communities. Depending on the land-use type, however, either soil or plant-surface bacteria were found to dominate the community. As the atmospheric bacterial community was distinct from its source communities, which was driven by the different relative abundances of bacterial taxa, the existence of a microbial community characteristic for the atmosphere was implied [3].

Diverse bacterial communities have been described in the atmosphere [4] and in clouds [5], [6]. However, bacterial communities in cloud water may be distinct from bacterial communities in the dry atmosphere, as the chances of airborne bacteria to enter into cloud droplets are increased for those that can act as cloud condensation nuclei [7]. After entering cloud droplets, bacteria are thought to influence physical and chemical processes in the atmosphere [1]. They may do this both by the means of their outer membrane structures as well as their metabolic activity. During their residence time in clouds, a group of mainly epiphytic Gram-negative bacteria could influence patterns of precipitation by facilitating the formation of ice crystals [8]. The so-called ice nucleation active (INA) bacteria are among the most efficient described ice nucleators. By forming large aggregates of INA proteins, which are anchored in their outer membrane [9], INA bacteria substantially elevate the freezing temperature of water. Thus, they may be important in mixed phase clouds, where subzero temperatures are often too high for water to freeze in the absence of ice nucleators.

There is also growing evidence that some cloudborne bacteria proliferate in cloud droplets. It was observed for two cloud events that the majority (72% and 95%) of cloud bacteria were viable [10]. Also, Hill et al [11] showed that on average 76% of cloudborne bacteria from two clouds were metabolically active. A couple of studies confirmed that the indigenous bacterial communities from rain- and cloud water could grow on either naturally present or supplemented organic compounds [12], [13]. Several isolates from clouds were shown capable of metabolizing nutrients present in cloud water [14] at rates that make them competitive with photooxidation [15]. However, it remains unclear whether cloud bacteria are in fact active *in situ*.

Inside storm clouds water droplets can coalesce into hailstones. During their formation, hailstones collect cloud and rain droplets in a non-selective way as they circulate inside the cloud, following unpredictable individual paths. We have recently shown that hailstones, which preserve the samples by freezing in real time, are useful sampling tools of storm cloud water and, indirectly, of air from the atmospheric boundary layer that has been sucked up by the storm cloud [6]. The storm cloud bacterial community was diverse with the estimated total bacterial richness of 1800 operational taxonomic unites (OTUs) at the species level and with a medium species evenness as estimated from Lorenz curves [6]. We also suggested that the highly diverse community encompasses strains with opportunistic ecologic strategy, which may grow despite the short residence times in clouds. Although some of the isolates have been characterized as opportunists [6], it remains unclear, whether the pool of organic chemicals can support the metabolism of these bacteria and if selective enrichment of some bacterial groups actually occurs in clouds. We report here on a comprehensive biogeochemical study, analyzing large hailstones from the same hail event [6]. By performing a detailed molecular characterization of water-soluble organic matter in hailstones and by aligning the potential substrates with the characteristic bacterial genera present in the cloud, we investigate the possibility of microbial growth in the storm cloud.

## Materials and Methods

### Ethics Statement

All sampling sites were public property and non-protected areas. In addition, in Slovenia there is no legal requirement for obtaining permits for taking precipitation samples. Thus, there were no specific permits required for the described field studies. Endangered or protected species were not in any way affected by or involved in the sampling activity.

### Collection and cleaning of the hailstones

Forty two hailstones were collected after a thunderstorm discharged over Ljubljana, Slovenia in the late afternoon of May 25th, 2009. Hailstones were collected into sterile bags within 5 minutes after they fell on ground and stored at −20°C. For molecular characterization and analysis of dissolved organic carbon (DOC) as well as total dissolved nitrogen (TDN), the surface of 18 hailstones was cleaned by rinsing with deionized water. Ice cubes of deionised water, with their surface contaminated by soil and grass, were treated in the same way as a control for the rinsing procedure. All plastic and glass lab ware was acid washed; all metal equipment used was treated by dry-heat-sterilization (160°C, over night). The cleaning of hailstones was done under conditions minimizing contamination by organic vapour.

For microbiological analysis, the surface of 24 hailstones was sterilized under sterile conditions as previously described [6]. For flow cytometry analysis 1.8 ml each of 12 hailstones was fixed in 2% glutaraldehyde, the remainder of these 12 hailstones was either refrozen, stored at −20°C, or used for the enumeration of colony forming units (CFU) using R2A plates [16].

### Determination of dissolved organic carbon (DOC) and total dissolved nitrogen (TDN)

DOC and TDN were analyzed in 18 hailstones by low-volume manual injection and catalytic high-temperature combustion on a Shimadzu TOC-V analyzer with a total nitrogen module (TNM-1) [17]. Samples were acidified to pH = 2 with HCl (p.a.) and purged for 10 minutes with synthetic air prior to analysis to remove inorganic carbon. The accuracy of the analysis was confirmed with deep-sea reference water samples provided by the University of Miami. The accuracy with respect to deep-sea water was within 5% relative error and detection limits were 5 µM for DOC and 1 µM for TDN. Procedural blanks did not yield detectable amounts of DOC and TDN. Eight controls for the cleaning procedure were analyzed in the same way as hailstones and showed significantly lower values than the hailstones (Mann–Whitney U test, W = 144, p<0.0001 for both DOC and TDN). The negative controls were used for blank-correcting DOC and TDN concentrations. As the data were not normally distributed (Shapiro-Wilk normality test, W = 0.4, p<0.0001 for both DOC and TDN), we report the median (Me) together with the quartile 1–quartile 3 values (Q1–Q3) and use a nonparametric test for the analysis of correlation (Spearman's rank correlation coefficient).

### Characterization of dissolved organic matter (DOM)

On three individual hailstones, covering the DOC concentration range, a detailed molecular characterization was performed using ultrahigh-resolution mass spectrometry on a 15 Tesla Bruker Solarix electrospray ionization Fourier-transform ion cyclotron resonance mass spectrometer (ESI-FT-ICR-MS). For FT-ICR-MS analysis, DOM was isolated from the hailstones via solid phase extraction [18]. DOM was directly infused into the mass spectrometer in methanol∶water (1∶1). The samples were ionized by electrospray ionization (ESI) in negative and positive mode. This ionization technique produces singly charged ions and keeps covalent bonds intact. 500 scans were accumulated in broad band mode for each sample. Procedural blanks did not contain detectable impurities. The mass spectra were internally calibrated. A mass error of <20 ppb was achieved for each detected mass. Based on this ultrahigh precision, molecular formulae were calculated for each peak. Programs used for data analysis and interpretation were Bruker Solarix Control, Bruker Data Analysis, Microsoft Access, and Ocean Data View. The difference between the three analyzed hailstones was insignificant compared to triplicate analysis of the same sample; therefore we discuss the average of the three samples.

### Total bacterial abundance

Total bacterial abundance was determined using a FacsCalibur flow cytometer (Becton Dickinson, Franklin Lakes, NJ) equipped with an air-cooled laser providing 15 mW at 488 nm employing a standard filter set-up. The samples were stained with SYBRGreen I (final concentration 0.02% of the stock solution, Molecular Probes Inc., Eugene, OR) for 15 min in the dark, at room temperature [19]. Fluorescent microspheres (Molecular Probes Inc.) with a diameter of 0.95 µm were analyzed as a standard. Sterilized ice cubes of deionized water were fixed and analyzed in the same way as the samples. The densities in the negative controls were significantly lower than the densities in hailstones (Mann–Whitney U test, W = 47, p<0.005). As the data were not normally distributed (Shapiro-Wilk normality test, W = 0.5, p<0.0001), we report the median (Me) and the quartile 1–quartile 3 values (Q1–Q3).

### The analysis of bacterial sources

The 16S rRNA gene sequences of the clones and the isolates, which have been previously reported under GenBank accession numbers JQ896628–JQ897350 [6], were analyzed for the community composition of individual hailstones. Operational taxonomic units (OTUs) were created by 99% similarity using the CD-HIT Suite: Biological Sequence Clustering and Comparison [20]. The Ribosomal Database Project (RDP) classifier was used for naive Bayesian classification of sequences [21]. The taxa that were independently sampled by at least 3 hailstones were considered characteristic and the presence of taxa only sampled by 1 or 2 hailstones was regarded as coincidental. The cultivable community was investigated on the genus level, whereas the total community was analyzed on the phylum, class or order level.

## Results and Discussion

**Figure 1:**
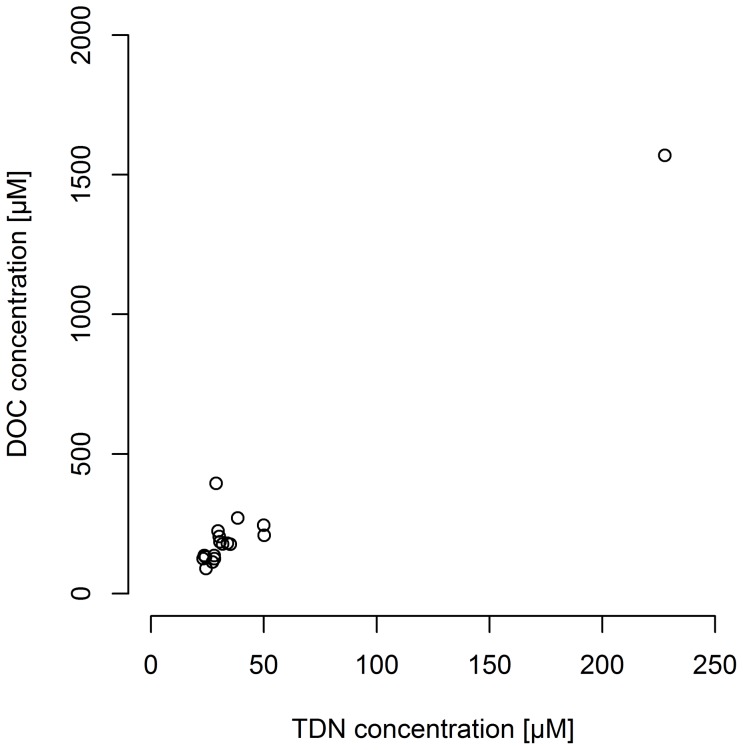
The correlation between DOC and TDN. Dissolved organic carbon (DOC) is presented as a function of dissolved total nitrogen (TDN). The DOC and TDN concentrations were significantly correlated (Spearman's rank correlation coefficient, rho = 0.749, p<0.001, n = 18).

The composition of dissolved organic matter (DOM) was determined in terms of quantity and quality. Our bulk analysis of dissolved organic carbon (DOC) and total dissolved nitrogen (TDN) in 18 hailstones revealed high concentrations of both DOC and TDN. Concentrations of DOC ranged between 90 and 1569 µM, with a median DOC concentration of 179 µM (Q1–Q3 = 132–220 µM). TDN concentrations ranged between 23 and 228 µM, with a median of 30 µM (Q1–Q3 = 27–35 µM). Similar DOC concentrations were previously reported for cloud water from orographic clouds [22] and rain [23]. The concentration of TDN was in the range of values reported for TDN in precipitation [24]. On average, more than two thirds of TDN was present as dissolved inorganic nitrogen (DIN) in the form of nitrate and ammonium [6]. It has previously been reported for precipitation in both rural and urban areas world-wide that inorganic nitrogen accounts for the major fraction of dissolved nitrogen [24]. Considering that the concentrations of DOC and TDN in storm clouds are within the same range as the concentrations measured in rivers, lakes and oceans [25], storm clouds can be classified as eutrophic environments. There was a significant correlation (Spearman's rank correlation coefficient, rho = 0.749, p<0.001, n = 18) between DOC and TDN concentrations (Figure 1), which suggests that carbon and nitrogen were derived from the same organic source, which either served as a condensation nucleus or got dissolved in cloud water. Subsequently, the source was diluted by deposition of water vapour or coalescence of other cloud droplets, causing the range of concentrations that we observed between individual hailstones. As most of the TDN in hailstones was inorganic, we assert that a mineralization process, involving photochemistry or biodegradation, took place after dissolution of the source organic compound into cloud droplets.

The ability of heterotrophic microorganisms to metabolize DOM is not only dependent on the quantity of DOM that is available, but also on the molecular composition of the DOM pool. Not all compounds may be equally degradable by the microorganisms that are co-occurring in the cloud droplets. Using ultrahigh-resolution mass spectrometry we characterized the molecular composition of DOM in three individual hailstones. FT-ICR-MS is the only method that allows obtaining molecular information on individual compounds in complex DOM mixtures. The method has been successfully used to get insights into the molecular composition of DOM in marine and in freshwater systems in hitherto unprecedented detail [26]. Here, we applied this advanced analytical technique for the first time on hailstones. Very small volatile organic compounds (<150 Da) escaped our analytical window. Their ubiquitous presence in the atmosphere has already been described elsewhere [22], thus we focused on molecules of the higher molecular mass range. The molecular formulae of 2839 compounds were identified. More than 99% of them were in the molecular mass range of 150–1000 Da. The median mass of all compounds was 354 Da. The median molecular formula was C_16_H_22_O_7_, i.e. half of the detected compounds contained more, and half less, of the respective element. The large molecular diversity and the molecular mass range of DOM in hailstones were comparable to DOM in aquatic systems [26] as well as to water-soluble compounds in aerosols [27]. Forty-four percent of all identified compounds contained one or two nitrogen atoms. All nitrogen was associated to phenolic and unsaturated compounds, whereas peptides and proteins were not present in detectable concentrations. While the molecular diversity of nitrogen-containing compounds was high (1242 compounds contained nitrogen), their abundance in terms of relative concentration was low. Thirteen percent of all compounds contained one sulfur atom. Most sulfur containing compounds were sulfonic acids, some of which are common synthetic products.

**Figure 2:**
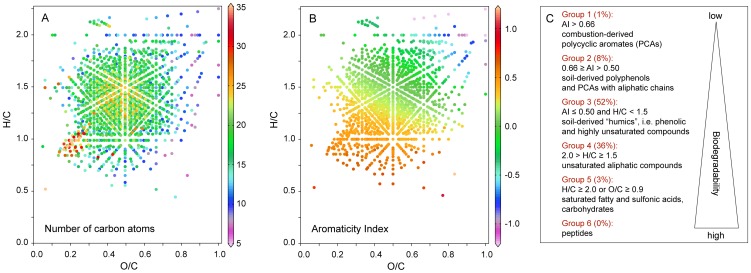
The molecular composition of dissolved organic matter in hail. The element ratio H/C is plotted as a function of O/C for each detected molecular formula detected by ultrahigh-resolution mass spectrometry (FT-ICR-MS) in at least one of the three hailstones. Each dot in these plots represents the molecular formula of an intact molecule. Panel A: The number of carbons in each molecular formula is displayed as a color code in the third dimension. Most compounds are large (C>10) and polar (O/C>0) and are likely not volatile. Panel B: The aromaticity index (AI-mod, [29]) of each molecular formula is displayed as a color code in the third dimension. An aromaticity index 0.5<AI is unambiguous evidence for aromatic compounds and an aromaticity index 0.66<AI is unambiguous evidence for condensed aromatics. Panel C: Compound groups were assigned to molecular formulae based on their aromaticity and element ratios [29], [39]. The biodegradability roughly increases from group 1 to group 6, e.g., polycyclic aromates are among the most stable compounds in the environment, whereas most peptides are quickly decomposed in the environment. Peptides (group 6) have the same characteristics as group 4, but contain nitrogen.

The molecular composition of higher molecular mass range DOM is indicative of its history. A few compounds contained less than 10 carbon atoms (Figure 2) and were potentially volatile, but most compounds were too large to be volatile and must have reached the atmosphere as particles. As aromatic compounds in dissolved organic matter are very susceptible to photochemical decay [28], the dominance of this compound class in the hailstones is indicative of a fast transfer from soil to atmosphere and hail on the time scale of less than one day.

The aromaticity of each molecular formula was assessed with help of the Aromaticity Index (AI) [29]. An aromaticity index 0.66<AI is an unambiguous criterion for polycyclic aromatic structures that are produced during combustion, but not by organisms. An aromaticity index 0.5<AI is an unambiguous criterion for the presence of aromatic compounds that are abundant in vascular plant debris (e.g. lignin). Thus compounds having an aromaticity index 0.5<AI<0.66 are plant derived material that has undergone microbial degradation in soils. By using AI and element ratios of molecular formulae (H/C and O/C), all detected molecules were grouped according to their molecular structure. The majority of the compounds (60%) were highly unsaturated or phenolic organic acids, typical for soil-derived organic matter in rivers (groups 2 and 3 in Figure 2) (e.g. [30], [31]). Less than 3% of the identified compounds were plant waxes, fatty acids or carbohydrates (group 5 in Figure 2), and less than 1% of the compounds were unambiguously combustion-derived (group 1 in Figure 2). As the majority of compounds were soil-derived (groups 2, 3 and 4 in Figure 2) and leaf surface compounds were present to a lesser degree (part of group 5 in Figure 2), the most likely scenario that explains the molecular composition of DOM in the hailstones is dissolution and desorption of organic matter from soil particles that were mobilized from a local source. A fraction of these particles most probably carried bacterial cells, which often are found aerosolized attached to particles [2]. Peptides were not present in detectable concentrations, thus the only highly biodegradable group of compounds were plant waxes, fatty acids and carbohydrates (group 5 in Figure 2), which represented less than 3% of the identified compounds. Due to the short lifetime of the storm cloud, from which the hailstones were obtained [6], only group 5 compounds are likely to be metabolized by bacteria during their residence time in the atmosphere. As 80% of the cloud water remains airborne after the hail event, the hailstones do not represent the eventual fate of all cloud water, containing organic matter and bacteria. When the cloud dissipates and the droplets evaporate, the aerosols remain airborne and can serve as condensation nuclei for future cloud events. Thus, the detected trace amounts of easily degradable organic compounds, may serve as bacterial substrate even after the cloud has dissipated.

**Figure 3:**
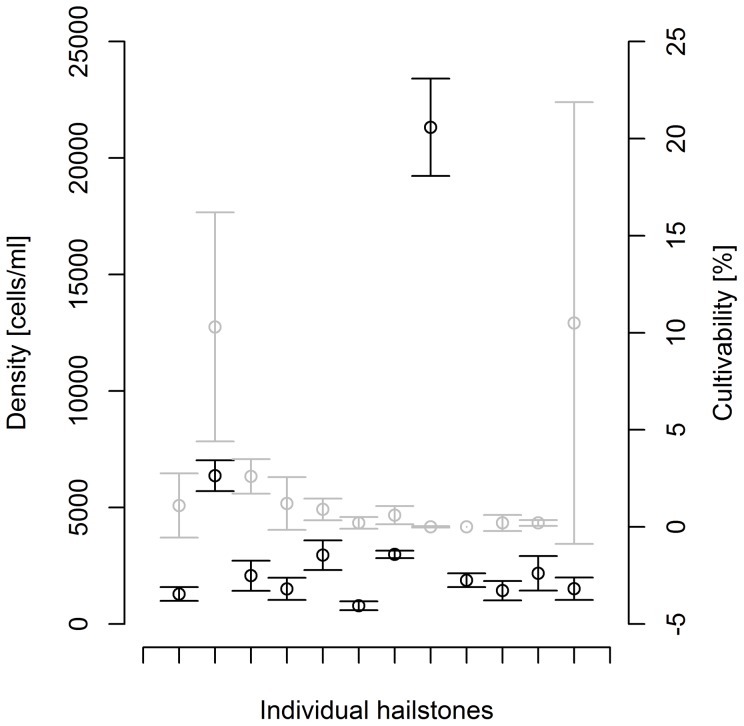
Bacterial density and proportion of cultivable cells. The mean density of bacterial cells as determined with flow cytometry in individual hailstones is presented (gray lines). The proportion of cultivable cells is shown for the same hailstones (dark lines). Error bars denote the standard deviation.

The analysis of 12 individual hailstones by flow cytometry revealed total bacterial numbers ranging from 778 to 21 321 cells per ml (Me = 1973, Q1–Q3 = 1485–2960, Figure 3). The bacterial densities in the storm cloud are in the lower range of previously reported cell numbers in cloud water, which ranged between 1500 [13] and 430 000 [11] bacteria per ml. Based on the average bacterial density in hailstones and an assumed initial cloud droplet diameter of 10 µm [32], we can calculate that on average only 1 out of 10^6^ storm cloud droplets carried a bacterial cell. Thus, cloud droplets are sparsely populated environments, where competition for nutrients and space between bacterial cells is likely insignificant. In addition, we can conclude that cloud water is a nutrient-rich microbial environment, in which significant increase in cell numbers would be possible even if only 3% of the high molecular mass DOM is readily biodegradable.

The median cultivability of bacteria was 0.8% (Q1–Q3 = 0.2%–1.5%), with high variability characteristic for individual hailstones. The reported range of cultivable bacteria in the atmosphere is between 0.01% and 75% [2]. However, cloudborne bacterial communities have previously been found to be characterized by a lower cultivability of between <1% and 2% [10], [33]. Up to 10.5% of all storm cloud bacteria were cultivable on nutrient agar plates (Figure 3), a property that is consistent with an opportunistic ecologic strategy. Lower cultivability of cells from some hailstones was probably a result of stress factors that the cells were subjected to during hailstone formation. E.g. these cells could have been subjected to several cycles of freeze-thawing during hailstone formation, which may cause cultivable bacteria to die or develop into a viable but non-cultivable state [34]. The fact than an unusually high cultivability has recently been described for epiphytic bacterial community [35], might point to an epiphytic origin of a part of storm cloud community [36], although high cultivability is not a strong proof by itself.

**Figure 4:**
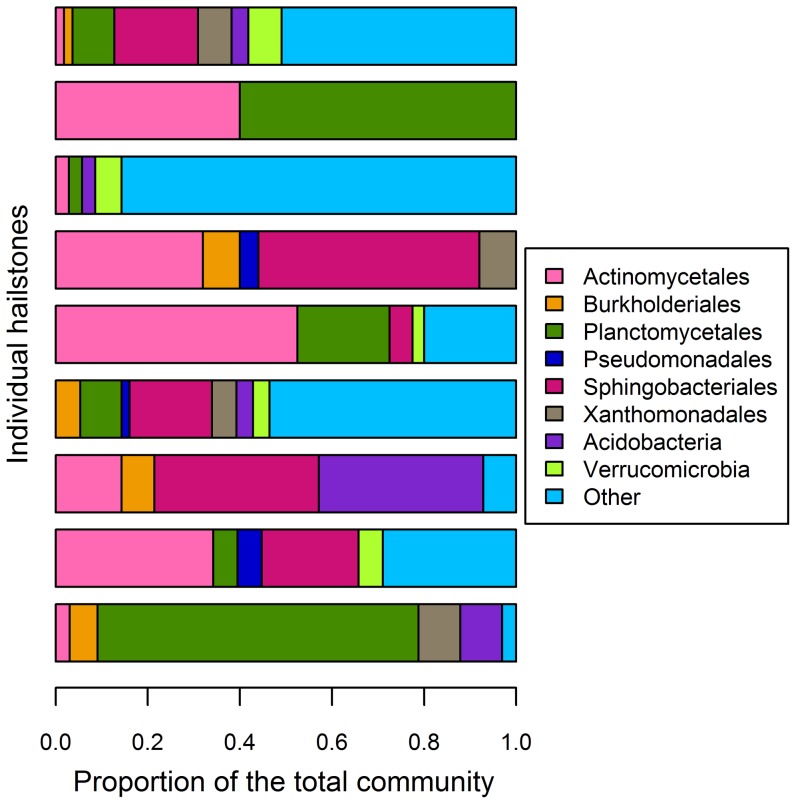
Total community composition in the storm cloud. Proportion of characteristic orders and phyla in 9 out of 12 hailstones, from which clone libraries were made. Characteristic orders and phyla are defined as the ones detected in ≥3 hailstones.

The total bacterial community was highly variable in individual hailstones (Figure 4), which is likely a consequence of storm clouds being a highly dynamic and temporary environment. However, there were taxa that were found in ≥3 hailstones and may represent typical cloud inhabitants. Bacterial orders and phyla, of which representatives were found in at least three individual hailstones, are termed characteristic and are presented in Figure 4. Sequences from *Actinobacteria* (23% of all sequences) were found in all investigated hailstones and representatives from *Plantomycetes* (11%), the *Bacteroidetes* (14%) as well as the γ-*Proteobacteria* (12%) were found in ≥5 hailstones. Also, sequences belonging to *Acidobacteria* (3%), *Verrucomicrobia* (3%), α- (5%) and β-*Proteobacteria* (8%) were present in ≥3 individual hailstones. Terrestrial habitats, and plants in particular, have been identified as major sources of bacteria in the atmosphere [2]. When investigating the spatial variability of airborne bacterial communities, Bowers et al [3] found that the community composition depended on the type of their source environment. Considering the high concentrations of *Actinobacteria* and *Bacteroides* and the low concentrations of *Rhizobiales* (Figure 4), the storm cloud community of our study resembles most closely the airborne communities found by Bowers et al [3] at suburban locations. This agreement fits well with the location of the storm cloud formation, which was over a city. The indicative bacterial taxa of our study pointed to a predominant terrestrial source of the atmospheric bacterial community [3]. The low abundance of *Acidobacteria* and *Rhizobiales* (Figure 4) indicated that soil bacteria were not dominant in the community, whereas the high relative abundance of γ-*Proteobacteria* and *Sphingobacteriales* (Figure 4) suggested that the microbiota in cloud droplets is to a large extent influenced by bacteria of epiphytic origin. This fits well with the high fraction of cultivable cells found for the storm cloud community and is consistent with the results obtained by others (e.g. [3]).

**Figure 5:**
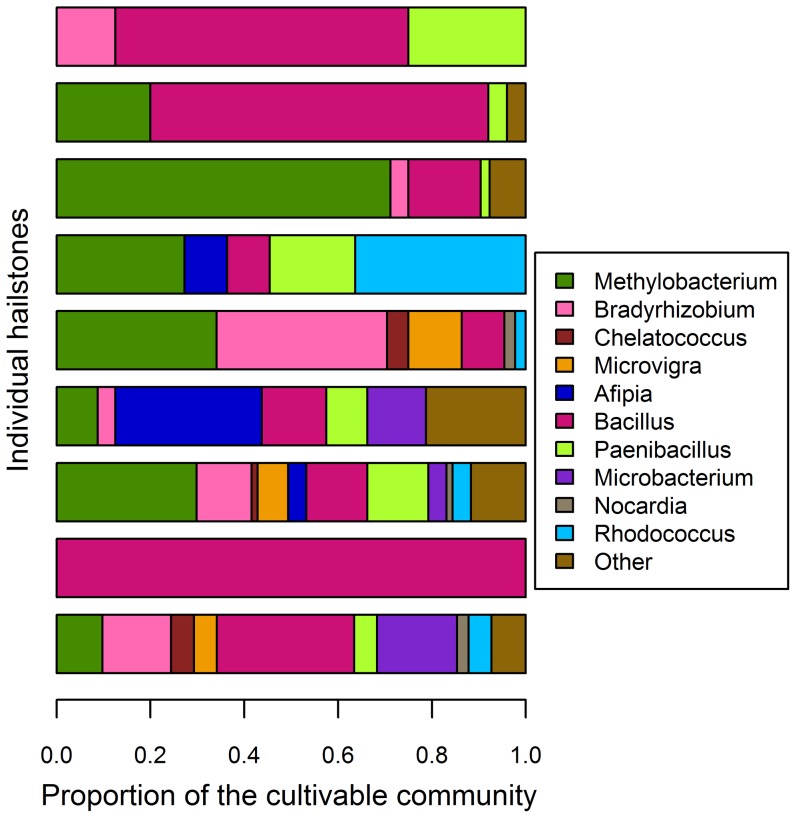
Cultivable genera in the storm cloud. Proportion of characteristic cultivable genera in 9 out of 12 hailstones, which contained cultivable bacteria. Characteristic genera are defined as the ones isolated from ≥3 hailstones.

Common cultivable genera, found in individual hailstones, are presented in Figure 5. Bacterial genera of the cultivable community, which were isolated from ≥3 individual hailstones, were considered characteristic for the cloud. In contrast to the community represented by the clone library, the cultivable bacterial community had a higher proportion (43.5%) of characteristic genera (Figure 5). Some of the characteristic genera (*Bacillus*, *Paenibacillus*, *Bradyrhizobium*) that were represented by isolates are consistent with soil origin of the cultivable community, but there was a remarkable (22%) contribution of typical plant-surface bacteria belonging to the genus *Methylobacterium*. They are adapted to a number of stress factors common for plant surfaces and the atmosphere [6], and therefore predestinated to remain active in the airborne state. We found, for example, that about 90% of the *Methylobacterium* isolates produced reddish, most likely carotenoid-type pigments, which can protect the cells against UV-induced cell damage [37]. In addition to being adapted to atmospheric stress, several *Methylobacterium* isolates have a wide substrate range, which is consistent with an opportunistic ecological strategy [6] and would predispose these cells to growth in the atmosphere. On the contrary, members of typical soil inhabiting genera, *Bacillus* and *Paenibacillus*, most likely get airborne as endospores, which hinders their growth in the atmosphere.

Despite the fact that the total and cultivable bacterial community composition as well as high cultivability all indicated the dominance of plant-associated bacterial groups, the molecular characteristics of DOM pointed to a soil origin of most aerosol particles in the cloud droplets. In fact, very few molecules suggested direct plant-surface origin, as the plant-derived compounds showed the chemical signature of decomposition in soil prior to aerosolization. A likely explanation for the discrepancy between chemical and microbial data is that bacteria originating from plant surfaces are better adapted to survival and growth in the atmosphere, whereas the stressors encountered in the atmosphere such as desiccation and UV radiation act as strong selective barriers against the soil inhabiting bacteria. Consequently, epiphytic bacteria may get enriched in the atmosphere, which could not only affect the chemical composition of the atmosphere, but also impact precipitation patterns more strongly than previously thought, as most INA bacteria stem from plant surfaces [38].

## Conclusions

The unique data sets that we obtained by analyzing individual hailstones provide us with unprecedented insight into the microbial and chemical inventory of storm clouds. They allow us to conclude that while the majority of aerosols were mainly soil-derived, the storm cloud contained microbial communities with a strong plant-surface signature, which links the troposphere to the phyllosphere. Many plant-associated bacteria are efficient in utilizing variable substrates on short timescales as well as in coping with atmospheric stress. Growth of these bacteria can be supported by the trace amounts of carbohydrates, lipids and some nitrogen-containing compounds that we detected among the high molecular mass DOM. The accumulating evidence strongly points to a selection process of bacterial cells in the course of cloud formation, which likely impacts the long-distance transport and the global distribution of bacteria. Our study on hailstones indicates that storm clouds are among the most extreme habitats on Earth, where microbial life can exist.
